# Isolation and Histopathological Changes Associated with Non-Tuberculous Mycobacteria in Lymph Nodes Condemned at a Bovine Slaughterhouse

**DOI:** 10.3390/vetsci7040172

**Published:** 2020-11-10

**Authors:** Angélica M. Hernández-Jarguín, Julio Martínez-Burnes, Gloria M. Molina-Salinas, Ned I. de la Cruz-Hernández, José L. Palomares-Rangel, Alfonso López Mayagoitia, Hugo B. Barrios-García

**Affiliations:** 1 Facultad de Medicina Veterinaria y Zootecnia, Universidad Autónoma de Tamaulipas. Cd. Victoria, Tamaulipas C.P. 87000, Mexico; jmburnes@docentes.uat.edu.mx (J.M.-B.); ncruz@docentes.uat.edu.mx (N.I.d.l.C.-H.); jlpalomares@docentes.uat.edu.mx (J.L.P.-R.); hbarrios@docentes.uat.edu.mx (H.B.B.-G.); 2 Unidad de Investigación Médica Yucatán, Unidad Médica de Alta Especialidad Hospital de Especialidades 1 Mérida, Yucatán, Instituto Mexicano del Seguro Social, CP 97150, Mexico; gloria.molinas@imss.gob.mx; 3 Department of Pathology & Microbiology, Atlantic Veterinary College, University of Prince Edward Island, Charlottetown, PE C1A4P3, Canada; lopez@upei.ca

**Keywords:** non-tuberculous mycobacteria, tuberculosis, granulomatous lesions, tuberculin test

## Abstract

**Background**: non-tuberculous mycobacteria (NTM) infect humans and animals and have a critical confounding effect on the diagnosis of bovine tuberculosis. The Official Mexican Standard (Norma Oficial Mexicana, NOM-ZOO-031-1995) for food safety regulates *Mycobacterium bovis* in cattle, but not the NTM species. The study’s objective was to isolate and identify the NTM present in condemned bovine lymph nodes in a slaughterhouse, characterize the histological lesions, and correlate bacteriological and microscopic findings with the antemortem tuberculin skin test. **Methods**: from 528 cattle, one or two pooled samples of lymph nodes from each animal were cultured for *Mycobacteria* spp. and processed for histopathology. **Results**: mycobacteria were isolated from 54/528 (10.2%) of the condemned lymph nodes; 25/54 (46.2%) of these isolates were NTM; 4 bacteriological cultures with fungal contamination were discarded. Granulomatous and pyogranulomatous inflammation were present in 6/21 (28.6%) and 7/21 (33.3%) of the NTM-positive lymph nodes, respectively. The species of NTM associated with granulomatous lymphadenitis were *M. scrofulaceum*, *M. triviale*, *M. terrae*, and *M. szulgai*, while those causing pyogranulomatous lesions were *M. szulgai*, *M. kansasii*, *M. phlei*, and *M. scrofulaceum*. **Conclusions**: the NTM infections can cause false-positive results in the tuberculin test because of cross immune reactivity and interference with the postmortem identification of M. bovis in cattle.

## 1. Introduction

Tuberculosis (TB) is one of the most devastating zoonotic diseases that, for centuries, has afflicted humans and animals worldwide [1,2]. A report by the World Health Organization indicates that over 1.5 million people die each year because of TB, and as many as 2 billion people are actively or silently infected [3]. The infection starts when humans or animals become exposed to genetically related species of mycobacteria, namely *Mycobacterium tuberculosis*, *M. bovis*, and the *M. tuberculosis* complex. These mycobacteria evade the immune and phagocytic systems and remain in infected organs for a long time, causing chronic inflammation, debilitating disease, and become a source of infection for other susceptible animals. For the last two decades, TB became an intrinsic part of the “One-Health” or “Global Health” perspective, where human and animal diseases are closely interconnected with the food and the environment [3]. 

Immunosuppression is a risk factor for TB and NTM infections [4,5]. Epidemiological studies demonstrated a relevant inter-species transmission of TB between cattle, non-bovine domestic species, and wildlife [6,7,8]. Transmission involving domestic and wild animals is a significant risk factor for cattle [9,10]. In the last decade, a transmission mode for NTM involving humans and animals has also been postulated [10]. The *Mycobacterium ulcerans* mode of transmission suggests that domestic animals can be infected when feeding in a contaminated environment, through simple contact with soil or feces of wild animals [11]. Humans can later be infected through direct contact with animal feces, animal bites, or ectoparasites [12,13]. In multi-host systems, sympatric hosts might also contribute to disease persistence and transmission [14,15].

Strategies to monitor and eradicate TB are underway in practically all countries around the world [3]. In Mexico, the national program for control and eradication of bovine TB started in 1990, and a few years later the guidelines for antemortem and postmortem diagnoses were streamlined under the Official Mexican Standard (Norma Oficial Mexicana, NOM-031-ZOO-1995). Antemortem detection of bovine tuberculosis is carried out using a dermal test that measures the immune response to mycobacterial antigens on the skin. For official certification, the following tests are used. The caudal fold tuberculin test (CFT) consists of the intradermal injection of 0.1 mL of purified bovine protein derivative (PPD) made with the strain AN5 of *M. bovis* in the caudal fold, and the cervical tuberculin test (CTT) is the intradermal injection in the mid-cervical region, with readings and measurements in millimeters, with a caliper at 72 h after injection. Subsequently, to evaluate the comparative cervical test, 0.1 mL of avian PPD made with the D4 strain of *M. avium* is injected intradermally in the mid-cervical area, and the reading is performed at 72 h. Animals infected with TB show a characteristic thickening of the skin at the site of inoculation.

The postmortem detection of bovine TB is accomplished by abattoir surveillance, where meat inspectors examine organs and lymph nodes for lesions compatible with TB. Affected or enlarged tissues are condemned and submitted to the laboratory for histopathological and bacteriological analyses. Microscopically, in tissues with TB, characteristic granulomatous lesions with the presence of intralesional acid-fast bacilli are observed. The bacteriologic diagnosis of TB is based on the identification of *M. bovis*, either by the bacteriological culture or by PCR. Farm quarantine and meat condemnation at the slaughter plant cause significant economic losses to farmers and producers [16].

One major problem for the antemortem and postmortem diagnosis of bovine TB is the confounding effect of non-tuberculous mycobacteria (NTM), widely disseminated in the environment. Infection with NTM produces a false-positive tuberculin test because these saprophytic bacteria cross-react with the immune response to the classical tuberculous mycobacteria [17,18,19,20]. In addition to the cross immune response, the gross and microscopic lesions induced by NTM are sometimes indistinguishable from those produced by *M. tuberculosis*, *M. bovis*, and the *M. tuberculosis* complex [17,18].

The NTM species most frequently isolated in South America and Central Africa from TB-like lesions in dairy and beef cattle are *M. fortuitum, M. szulgai*, *M. celatum*, *M. terrae*, *M. nonchromogenicum*, *M. intracellulare*, and *M. gordonae* [21,22,23]. The confounding effect of NTM in the antemortem and postmortem diagnosis of bovine TB is of significance in surveillance and meat inspection.

The study aimed to characterize the histological lesions in bovine lymph nodes condemned in the slaughterhouse, identify the species of NTM cultured from these nodes, and, finally, correlate the histopathological and bacteriological findings with the tuberculin tests.

## 2. Materials and Methods 

### 2.1. Collection of Lymph Nodes 

As part of surveillance for the national program for control and eradication of bovine TB in Mexico, lymph nodes were condemned by certified veterinary inspectors from 528 animals, at an abattoir in Tamaulipas, between 2007 and 2011. A single or two-pooled samples of the condemned lymph nodes were submitted to the laboratory for microbiological culture and histopathology. Antemortem diagnosis of TB in animals was based on immunological response to an intradermal PPD AN5 and D4 inoculation. Lymph nodes were cut serially and examined for gross lesions compatible with TB. One-half of the lymphoid tissue was preserved in 6% sodium borate for bacteriological analyses while the other half was immersed in 10% buffered formalin for histological examination. The specimens were sent to the Diagnostic Laboratory of the University of Tamaulipas and processed according to the Official Mexican Standard (Norma Oficial Mexicana, NOM-031-ZOO-1995).

### 2.2. Mycobacterial Isolation and Biochemical Identification

All samples were handled and processed in laminar-flow hood type II following established guidelines. After removing the sodium borate, the lymph nodes were placed in a sterile mortar where the fat was removed; the tissue was cut into small fragments and manually macerated with sterilized sand. Twenty mL of distilled, sterile water was added to the specimen for a second maceration. The macerate was then diluted with a 4% solution of sodium hydroxide and phenol red at a 1:1 ratio, and incubated at 37 °C shaking for 20 min. The suspension was centrifuged at 1157.13× *g* ×20 min, and, after removing the supernatant, the sediment was neutralized with a 1N hydrochloric acid solution until the pink color of the indicator turned yellow, as detailed in the Petroff protocol [24]. 

Two Stonebrink media tubes (PRONABIVE, México City, Mexico) and one Lowenstein–Jensen culture media tube (PRONABIVE, México City, Mexico) were inoculated with the neutralized residue and incubated at 37 °C in 5% CO_2_ for nine weeks, or until typical mycobacterial colonies were observed and macroscopically characterized. Mycobacteria was first confirmed by the microscopic observation of acid-fast bacilli (AFB) using the Ziehl–Neelsen stain. Mycobacterial cultures were evaluated for metabolic activity, niacin production, and nitrate reduction, catalase production at 22 °C and 68 °C, urease production, and hydrolysis of Tween 80. Bacterial growth and tolerance were also assessed in MacConkey agar without violet crystal and 5% sodium chloride.

### 2.3. Histopathology

Formalin-mixed lymph nodes were processed, embedded in paraffin, and cut at 4–5 µm using standard methods. Tissue sections stained with hematoxylin–eosin were microscopically evaluated for histologic lesions, and Ziehl–Neelsen stained tissues were assessed for acid-fast bacilli.

## 3. Results

### 3.1. Collection of Lymph Nodes and Gross Changes

From 528 animals with condemned lymph nodes, 235 (44.5%) showed gross lesions compatible with TB and 42/528 (8%) of these lymph nodes were from cattle that had tested positive for tuberculin (Table 1).

### 3.2. Mycobacterial Isolation and Biochemical Identification

Mycobacteria were isolated from 10.2% (54/528) of the lymph node samples; 53.7% (29/54) of the mycobacterial isolates were identified as *M. bovis* and 46.2% (25/54) as NTM. Biochemical typing for 21 NTM isolates yielded *M. szulgai* (n = 8), *M. scrofulaceum* (n = 4), *M. phlei* (n = 2), *M. kansasii* (n = 2), *M. chelonae* (n = 2), *M. triviale* (n = 1), *M. fortuitum* (n = 1), and *M. terrae* (n = 1); 3 NTM isolates were discarded because fungal contamination precluded biochemical typing, and, in one case, species identification was not possible. 

Incidentally, 20.0% (5/25) of the lymph nodes harboring NTM were from animals that tested positive for the tuberculin test, and 4% (1/25) were from herds regarded as exposed, based on tuberculin tests.

### 3.3. Histopathology

Histopathologically, the 21 lymph nodes positive for NTM had two distinct morphologic types of inflammation: (1) granulomatous, characterized by an external rim of fibroblasts that contain macrophages, giant cells, central caseous necrosis, mineralization, and scant neutrophils; (2) pyogranulomatous inflammation, which—besides macrophages—giant cells, necrosis, and mineralization also observed abundant neutrophils. Granulomatous and pyogranulomatous inflammation were observed in 28.6% (6/21) and 33.3% (7/21) of the NTM-positive lymph nodes, respectively. Necrosis and mineralization were only present in 28.6% (6/21) of the lymph nodes. Two lymph nodes 28.6% (2/7) with pyogranulomatous inflammation also had intralesional fungi. AFB were microscopically observed in 83.3% (5/6) of the lymph nodes showing granulomatous inflammation, and in 57.1% (4/7) with pyogranulomatous inflammation. Seven 33.3% (7/21) lymph nodes yielded NTM, but did not have any microscopic lesions or changes associated with TB (Table 2). 

The species of NTM associated with granulomatous lymphadenitis were *M. scrofulaceum, M. triviale, M. terra*e, and *M. szulgai*, while those related to pyogranulomatous lesions were *M. szulgai, M. kansasii, M. phlei*, and *M. scrofulaceum*. However, the NTM species, except for *M. fortuitum* and *M. chelonae*, were also isolated from lymph nodes with no microscopic lesions. Interestingly, *M. scrofulaceum* and *M. szulgai* were isolated from lymph nodes with granulomatous and pyogranulomatous inflammation, as well as from those without microscopic changes (Table 2). 

Five animals were positive at the antemortem intradermal tuberculin test, and another animal belonged to a herd with positive reactors to tuberculin, which was considered “exposed.” One tuberculin-positive animal with granulomatous lesions yielded *M. terrae*. The live animal deemed as “exposed” had pyogranulomatous inflammation and was culture-positive to *M. scrofulaceum*. Three tuberculin-positive animals clearly did not actively show tuberculous lesions, but yielded *M. szulgai*, *M. phlei,* and *M. chelonae*. On the other hand, *M. kansasii* was isolated from a tuberculin-positive animal, but tissues were not available for histopathology.

## 4. Discussion

### 4.1. Species of NTM Isolated from Lymph Nodes 

The species of NTM isolated from the lymph nodes condemned by the meat inspectors at the slaughter plant in Tamaulipas were *M. szulgai, M. scrofulaceum, M. phlei, M. kansasii, M. chelonae, M. terrae, M. triviale,* and *M. fortuitum.*

In a review study of epidemiology of the human pulmonary infection with NTM, *M. avium* complex was the most common species isolated in North America, followed by *M. abscessus*/*chelonae*, *M. xenopi*, *M. fortuitum*, and *M. kansasii* [4]. Our findings differ from the results reported in a similar study in the south region of the State of Mexico where *M. neoaurum, M. parafortuitum, M. moriokaense,* and *M. confluentis* were the primary mycobacterial isolates [16]. Zaragoza et al. (2017) [16], using molecular markers present in the 23S rRNA gene and sequencing the 16S rRNA gene, identified the mycobacteria species in milk and nasal exudates of dairy cattle. However, it should be noted that our mycobacteria were identified by culture and came from the lymph nodes of beef cattle. Moreover, in our study, the biochemistry analysis used to identify mycobacteria could not accurately reflect the mycobacterial species. Therefore, our findings require further molecular analyses to support species identification. Nevertheless, the species of NTM reported here agree with those previously reported in Mexico in human lungs [25,26,27,28], water [29,30], and salad samples [31].

To date, the number of NTM species reported in the literature exceeds 130. According to some reports, the most common NTM isolates from cattle are *M. gastri, M. flavescens, M. phlei, M. triviale, M. terrae, M. nonchromogenicum, M. intracellulare, M. gordonae, M. thermorresistible, M. xenopi, M. fortuitum, M. chelonae, M. ulcerans and M. kansasii, M. avium, M. neoaurum, M. confluentis*, and *M. vaccae* [1,16,22,32,33,34,35,36,37]. It could be surmised from these findings that the environment in northern Mexico, and other regions in America, Europe, Asia, and Africa share the same species of saprophytic mycobacteria. 

Tuberculous bacteria disseminate by lymphatic drainage, making the lymph nodes the prime anatomical site for meat inspection and culture. Most of the lymph nodes in our study were mesenteric and mediastinal, indicating that the main port of entry for NTM is through the lungs and digestive systems [38]. It should be noted that 33.3% of lymph nodes without lesions, or changes associated with TB, tested positive for NTM, suggesting that lymphoid tissues harbored mycobacteria without eliciting inflammation, or that sufficient time had not elapsed after infection to trigger a microscopic inflammatory response. 

### 4.2. Types of Inflammation

Enlargement of a lymph node, clinically referred to as lymphadenomegaly, is the “red flag” that prompts meat inspectors to carefully examine these tissues and look for inflammatory reactions, such as granulomas or abscesses. Histopathological and microbiological studies are indispensable to define the lesions and identify the NTM organisms correctly. The host response elicited by mycobacteria is typically granulomatous, regardless of the bacterial species or the affected organ [39]. This granulomatous inflammatory response relates mainly to the lipids in the bacterial cell wall and the mycobacteria’s ability to overcome phagocytosis [39]. The deposition of mineral (calcium and phosphates) is frequent in tissues undergoing cell necrosis and chronic inflammation. This type of calcification, known as dystrophic calcification, tends to increase with time, and is not a specific lesion, since it occurs in many diseases other than tuberculosis [40].

It remains unclear, however, why *M. scrofulaceum*, *M. triviale*, *M. terrae*, and *M. szulgai* caused granulomatous inflammation, while *M. scrofulaceum*, *M. kansasii*, *M. szulgai,* and *M. phlei* caused a pyogranulomatous response. According to the literature, granulomatous and pyogranulomatous inflammation could be merely different stages of the inflammatory process, where pyogranulomas are less chronic and active, and granulomas more chronic and better organized [39]. Some studies with bovine lymph nodes, liver, and lungs reported that *M. fortuitum, M. avium, M. kansasii, M. lentiflavum,* and *M. intracellulare* were the main species associated with chronic inflammatory response and typical tuberculous granuloma formation [34,35,36,41]. Nonetheless, from the histopathological point of view, the lesions caused by NTM species are indistinguishable from *M. tuberculosis* complex [42]. 

The role of neutrophils in the pathogenesis of mycobacterial lesions is only partially understood. It has been observed in experimental mycobacterial infections that neutrophils favor the recruitment of other cells involved in forming granulomas [43]. The permanence of neutrophils at the site of injury depends on the production of chemical mediators, such as TNF-α [44]. Nonetheless, recent studies associate it mostly with interleukin-8 (IL8) [45], a potential marker for *M. bovis* infections [46]. Neutrophils arrive in two “waves”, the first relates to the innate response against the pathogen and the second to the adaptive response [47]. As a result, neutrophils can be present in the initial host response and remain in lower numbers in chronic granulomatous inflammation. 

Pyogranulomatous lymphadenitis occurs in cattle and black deer infected with *M. kansasii* [17,48]. This bacterium has also been isolated from left bronchial and caudal mediastinal lymph nodes from cattle [36]. 

Granulomatous lymphadenitis is not unique to mycobacterial infections since it also occurs with fungal and other bacterial infections. *Coccidioides immitis* and the algae *Chlorella* sp. are two systemic mycoses endemic in northern Mexico, which are important impersonators of TB. Both of these infections cause granulomatous and pyogranulomatous lymphadenitis in ruminants grossly indistinguishable from mycobacterial diseases [49,50]. In this sense, granulomatous lymphadenitis also has been described in red deer (*Cervus elaphus*) for apparent coinfections of *Corynebacterium pseudotuberculosis*, *M. bovis*, and *M. avium* subsp. *paratuberculosis* [51]. 

In this study, none of the culture tubes had colonies of different morphology. Our findings indicate that none of the isolated NTM species was part of a coinfection with organisms of the *M. tuberculosis* complex or another NTM species. However, there is not sufficient evidence to confirm the absence of bacterial coinfections conclusively. In the last decade coinfections of *M. bovi*s and *M. avium* subsp. *paratuberculosis* in cattle (*Bos taurus*) and wildlife have been documented [52,53,54,55]. However, it is also well known that simultaneous mycobacterial coinfection interferes with the accurate diagnosis of bovine TB [52,54,55], because a concurrent infection interferes with the diagnosis of bovine TB [52,54,55].

Moreover, NTM samples collected under field conditions are more likely to interfere with the intradermal diagnostic test for bovine TB [53]. Our findings and those reported in two studies [36,37] suggest that *M. terrae, M. kansasii, M, szulgai, M. scrofulaceum, M. phlei, M. chelonae, M. engbaekii, M. arupense, M. nonchromogenicum, M. heraklionense,* and *M. persicum* interfere in the antemortem intradermal tuberculin test for bovine TB leading to false-positives and considerable economic losses [18].

The cross-reactivity in cattle infected with *M. bovis* and NTM is a serious problem for TB surveillance and explains why the lymph nodes of few tuberculin-positive animals yielded NTM but not *M. bovis*. This cross-antigenic reaction results from the close phylogenetic relationship shared by NTM and *M. tuberculosis* [19,20]. NTMs and *M. bovis* belong to the same genus and have close phylogenetic homology, but can be differentiated by rRNA amplification [16]. 

Finally, lymph node enlargement is not unique to infectious diseases; it often occurs in neoplastic diseases, particularly lymphosarcoma prevalent in cattle worldwide [56]. None of the condemned specimens submitted to the laboratory in Tamaulipas had microscopic evidence of bovine lymphosarcoma or any other neoplastic disease.

## 5. Conclusions

Based on our findings and similar reports in the literature, it is abundantly clear that NTM infections can cause false-positive results in the tuberculin test because of cross immune reactivity, and also interfere with the postmortem identification of *M. bovis* in cattle. Careful examination of lymph nodes at slaughter and laboratory testing should be routine in meat inspection.

## Figures and Tables

**Table 1:** Total of samples, tuberculin skin tests, gross lesions, and number of mycobacteria isolated.

Year	Total of Samples	Tuberculin Reactors	Gross Lesions Compatible with TB	*M. bovis* Isolated	NTM Isolated
2007	109	11	43	8	5
2008	169	13	69	12	6
2009	19	8	8	2	1
2010	139	2	67	3	5
2011	92	8	48	4	4
Total	528	42	235	29	21

TB: Tuberculosis; NTM: Non-Tuberculous Mycobacteria.

**Table 2:** Association between microscopic findings and species of mycobacteria isolated.

Number of Samples	%	Microscopic Changes	NTM Isolates
6	28.6	Granulomas	*M. scrofulaceum, M. triviale, M. terrae, M. szulgai.*
7	33.3	Pyogranulomas	*M. szulgai, M. kansasii, M. phlei, M. scrofulaceum.*
7	33.3	Without changes	*M. szulgai, M. phlei, M. chelonae, M. fortuitum, M. scrofulaceum.*
1	4.8	Unknown	*M. kansasii.*
